# Stand Up and Fight Falls: Can a Video Intervention Help Reduce Falls in the Geriatric Population?

**DOI:** 10.7759/cureus.11508

**Published:** 2020-11-16

**Authors:** Margot Samson, Kathleen Davenport, Caroline Rizzo, Shan W Liu

**Affiliations:** 1 Emergency Medicine, University of Central Florida College of Medicine, Orlando, USA; 2 Emergency Medicine, University of North Carolina Hospital, Chapel Hill, USA; 3 Emergency Medicine, University of North Carolina School of Medicine, Chapel Hill, USA; 4 Emergency Medicine, Massachussetts General Hospital, Boston, USA; 5 Emergency Medicine, Massachusetts General Hospital, Boston, USA; 6 Emergency Medicine, Harvard Medical School, Boston, USA

**Keywords:** falls, geriatric, survey

## Abstract

Introduction: Falls are a major source of morbidity and mortality in the geriatric population. However, efforts to reduce falls have had limited success. This study examines if a video intervention presented in the ED to patients who have fallen could improve fall education and reduce future falls.

Methods: Patients 65 years and older who presented to a large academic ED for a fall between June and December 2017 were identified via triage note for an intercept study. Patients who did not speak English, who were cognitively impaired, or whose condition was too acute (determined by providing physician) were excluded. Sixty-two eligible and consenting patients were shown a six-minute video intervention with recommendations to prevent future falls. Primary objectives include (1) whether patients found the recommendations reasonable to implement and (2) rate of implementation. Secondary aims were (3) perceived health status between patients who followed the recommendations versus those who did not and (4) rates of recurrent falls and ED revisits between the two groups. Data were analyzed using the Newcombe-Wilson Score Method and Fisher's exact two-tailed t-tests.

Results: Of 62 patients enrolled, 38 were retained at a six-month follow-up. Ninety-two percent of patients found the video intervention to be a reasonable education tool. At six months, 44.7% of patients implemented behavioral changes discussed in the video, and 21.1% had at least one new fall, with no significant difference between people who implemented video interventions and those who did not (23.5% and 19.0%, difference 0.045, 95% CI [-0.24 to 0.34], p=1.0). The rate of return to the ED at six months for all patients was 31.6%, with no significant difference between the two groups (23.5% versus 38.1%, difference 0.146, 95% CI [-0.18 to 0.43], p=0.49). Difference in the proportion of people feeling the same or better between the two groups was not significant at either the one-month (66.7% versus 75.0%, difference 0.083, 95% CI [-0.21 to 0.34], p=0.75) or six-month follow up (64.7% versus 47.6%, difference 0.171, 95% CI [-0.17 to 0.46], p=0.34).

Conclusion: This study found that while most patients find behavioral interventions feasible and reasonable to implement, only half actually make changes to their lives to reduce the risk of falling. This suggests that identifying and limiting barriers to implementation should be a priority in future studies, along with exploring the relationship between interventions and health status, ED revisits, and recurrent falls.

## Introduction

Falls are a major source of morbidity and mortality in the geriatric population and a frequent chief complaint of patients presenting to the ED. Adults 65 years and older visit the ED more than any other age group, and the rate of those visits is increasing faster than other age groups [1]. These patients have longer lengths of stay, higher rates of admission, and greater resource utilization than other age groups [2]. For example, in 2015, the estimated medical cost of falls (both fatal and non-fatal) was $50 billion [3]. Of those geriatric patients who visit the ED, it has been reported that 33% have fallen at least once in the past year. Among those, rates of recurrent falls are even higher, recorded at 69% [4].

Many screening tools exist designed to identify risks of future falls and provide interventions for patients at increased risk [5,6]. Some of the risk factors that increase the risk of falling include polypharmacy, osteoporosis, self-reported depression, dementia, non-healing foot sores, functional mobility, and a history of previous falls [5-8]. Falls can occur anywhere and at any time, but are most common outside, in the winter, and in the afternoon [9].

Despite the identification of these risk factors, efforts to reduce the number of falls in the geriatric population have had limited success. One study found that patients believe their fall was caused by the environment, or by their own inattention, and did not recognize the multifactorial nature of falling. This same study also demonstrated that 41% of participants were “not concerned” about their risk of falls or recurrent falls [10]. In another study, 85% of fall patients reported that their fall was preventable [9]. Some studies show that patients in the community largely find fall prevention information “unnecessary, irrelevant, or inappropriate” [11]; however, another study conducted in the ED reported that patients believe that interventions based on “advice and counseling” would be welcome and effective [10]. This creates a unique opportunity for ED providers who can use the patient encounter as a teachable moment, especially if short, impactful interventions can be designed and implemented [12]. However, no study has been conducted to see whether an ED falls video would be a feasible ED fall prevention tool.

With funding from the American College of Emergency Physicians, we created a short video aimed at educating geriatric ED fall patients, thus capitalizing on that teachable moment. We conducted a pilot study to see whether this video intervention shown in the ED to patients who had recently fallen could be used to educate patients on fall reduction strategies and change their behavior to prevent recurrent falls. The primary aims of this study were to explore whether patients found the interventions reasonable to implement (aim one) and whether they implemented the suggested interventions once discharged (aim two). The secondary aims were to look at differences in self-reported health status between those who implemented changes after viewing the video and those who did not (aim three), and determining if educating the patients while in the ED for a fall decreased ED revisits or recurrent falls (aim four). Patients were interviewed in the ED and at one- and six-months with phone call follow-ups. As this is a pilot study, the aims were focused more on measuring trends in behavior changes, gauging the feasibility of the study tools and methods, and guiding future study designs rather than drawing any definitive conclusions on patient behaviors.

## Materials and methods

Participants

A convenience sample of participants was recruited for an intercept study in an urban, level one trauma, academic hospital ED with more than 100,000 annual visits [13]. Patients triaged to lower acuity areas and the observation units were eligible. Patients with a chief complaint of “fall” on the electronic medical record (EMR), or with a nursing triage note mentioning a fall, were identified and selected. Participants were recruited between 11 am and 7 pm Monday through Friday from June to December 2017, based on research assistant availability. Patients were eligible if they had fallen in the past two weeks (as noted in the chief complaint or ED triage note) and if they were 65 years or older. Patients who did not speak English (given the video was in English), whose condition was too acute or unstable, who declined to consent, or who were cognitively impaired (as determined by the provider for that patient) were excluded.

Protocol

**Figure 1:**
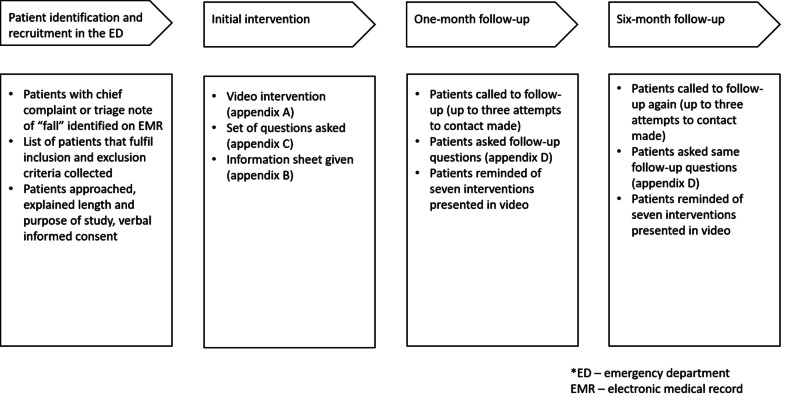
Flow diagram of the study procedure

The investigators designed and filmed a six-minute video called “The Seven Step Fall Challenge” (Appendix A). It includes seven recommendations to avoid future falls based on the STEADI (Stopping Elderly Accidents, Deaths and Injuries) Centers for Disease Control and Prevention Falls Prevention Guidelines, which focus on improving strength and balance, home safety, avoiding high-risk medications and dehydration, wearing appropriate footwear, and ways to prevent injury if you fall. Via the ACEP grant, the video was put online for easy reference and downloaded onto a tablet for convenient viewing by patients during the study. Eligible patients were approached by a research assistant and explained the purpose of the research study. Verbal consent was obtained from each patient, and each patient was also given a written information sheet explaining the purpose of the study and including relevant contact information for reference (Appendix B). Consented patients were then shown the falls video by the research assistant while in the ED and asked several questions after viewing the video (Appendix C). Participants were offered headphones to listen to the video if they preferred. The participants’ answers to these questions were recorded manually. In addition, study data were collected and managed using REDCap electronic data capture tools hosted at the hospital [14,15]. REDCap (Research Electronic Data Capture) is a secure, web-based software platform designed to support data capture for research studies providing: an intuitive interface for validating data capture; audit trails for tracking data manipulation and export procedures; automated export procedures for seamless data downloads to common statistical packages; and procedures for data integration and interoperability with external sources. Follow-up phone calls were conducted by the research assistant at one month and six months after initial enrollment (Appendix D). Patient demographics, including age, sex, race, and contact information was collected from the EMR after the patient’s consent. Research assistants were trained in how to approach a patient, obtain consent, show the video, and ask follow-up questions in a consistent manner to limit variability between patients. A flow diagram of the procedure is depicted in Figure 1.

Data analysis

For data analysis, Excel was used to calculate percentages for each demographic recorded, and a Fisher's exact two-tailed t-test analysis was run to determine significance between different study groups. Confidence intervals for differences in proportion were calculated using the Newcombe-Wilson Score Method. Cross tabulations were also performed to explore relationships between fall rates and health status based on whether recommendations were implemented or not.

## Results

Table 1 outlines the demographics of the study population. Sixty-two total people consented to participate in the study, with 47 of them retained for the one-month follow-up phone call and 38 of them for the six-month follow-up call. Patients had a mean age of 77 years, most were female, Caucasian, had high school education level, were married, and lived at home.

**Table 1:** Demographics n=62 ^a^Includes syncope, altered mental status, dyspnea, headache, head injury.

Demographic	Percentage of participants
Age, mean (years)	77 years (standard deviation +/- 7.9)
Men (%)	30.6
Caucasian (%)	93.6
Education (%)	-
Less than high school	9.7
High school, general education diploma	35.5
Specialty school	6.5
Two-year college	4.8
Four-year college	12.9
Masters/doctorate	16.1
Unknown	14.5
Marital status (%)	-
Married, civil union	41.9
Single	27.4
Divorced	4.8
Widowed	24.2
Living situation (%)	-
Home	88.7
Assisted living	3.2
Senior housing	8.1
Hospice	0
Chief complaint (%)	-
Fall	85.5
Pain	4.8
Other^a^	9.7

**Figure 2:**
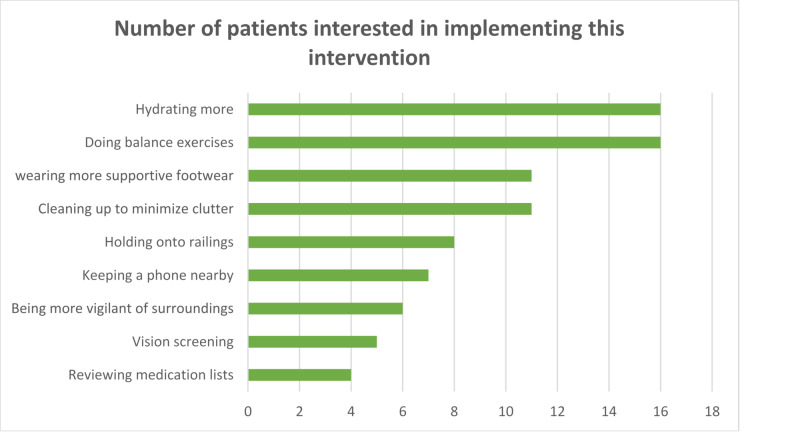
How will you follow/implement any of the recommendations in the video? n=62

Table 2 and Figure 2 summarize the participant responses to questions (Appendix C) after viewing the video. Ninety-two percent of participants found the recommendations in the video reasonable to implement. Answers to each question varied: common responses to “How will you follow/implement any of the recommendations in the video?” included hydrating more (n=16), exercising with a focus on balance training via tai-chi or yoga (n=16), wearing more supportive footwear, like sneakers instead of sandals (n=11), cleaning up around the house to limit clutter (n=11), holding on to railings (n=8), keeping a phone nearby to call for help (n=7), being more vigilant of the surroundings and taking more time to do things (n=6), checking up on vision (n=5), and reviewing medication lists (n=4). Responses were not all positive, however, as 16 patients mentioned that the recommendations were unnecessary, either because they already knew everything or because they did not find the information relevant to them. A visual representation of this data is shown in Table 2 and Figure 2 below.

**Table 2:** Views on video recommendations n=62

Are the recommendations in the video reasonable to implement? (aim 2)	-
Yes (%)	92.0%
No (%)	8.0%
How will you follow/implement any of the recommendations in the video? (aim 3). Percent not additive; patients can answer with more than one implementation	-
Increasing hydration (%)	25.8%
Improving balance (%)	25.8%
Improving footwear (%)	17.7%
Cleaning up the house (%)	17.7%
Using railings (%)	12.9%
Keeping a phone nearby (%)	11.3%
Slowing down (%)	9.7%
Vision check (%)	8.1%
Reviewing medications (%)	6.5%
“I already do it/ not relevant” (%)	25.8%

The study also looked at how many patients implemented the interventions (aim two), and whether they had recurrent falls or ED revisits since viewing the video (aim four). Approximately half of the participants stated that they implemented the interventions described in the video. At six months, 21.1% of all participants described having at least one new fall within six months of initial contact. The difference in incidence of new falls between patients who implemented video interventions and patients who did not was not statistically significant either at the one month mark (22.2% versus 15.0%, difference 0.072, 95% CI [-0.20, 0.31], p=0.71) or the six months mark (23.5% and 19.0%, difference 0.045, 95% CI [-0.24, 0.34], p=1.0). The rate of return to the ED at six months for all patients was 31.6%. The difference in rates of return to the ED between patients who implemented the interventions versus those who did not was not statistically significant at either the one month mark (7.4% versus 10.0%, difference 0.026, 95% CI [-0.18, 0.27], p=1.0) or six month mark (23.5% versus 38.1%, difference 0.146, 95% CI [-0.18, 0.43], p=0.49). The one-month (Table 3) and six-month (Table 4) results are shown below.

**Table 3:** Rate of falls and change of behavior after watching video – one-month follow-up n=47 ^a^95% CI

-	Implemented behavior change (57.4%)	Did not implement behavior change (42.6%)	Difference between those who implemented and those who did not	p-Value for the difference^a^
New falls (%)	22.2%	15.0%	7.2% [-0.20, 0.31]	0.71
Return to ED (%)	7.4%	10.0%	2.6% [-0.18, 0.27]	1.0
Health status: Improved or stayed the same (%)	66.7%	75.0%	8.3% [-0.21, 0.34]	0.75

**Table 4:** Rate of falls and change of behavior after watching video – six-month follow-up n=38 ^a^95% CI

-	Implemented behavior change (44.7%)	Did not implement behavior change (55.3%)	Difference between those who implemented and those who did not	p-Value for the difference^a^
New falls (%)	23.5%	19.0%	4.5% [-0.24, 0.34]	1.0
Return to ED (%)	23.5%	38.1%	14.6% [-0.18, 0.43]	0.49
Health status: Improved or stayed the same (%)	64.7%	47.4%	17.1% [-0.17, 0.46]	0.34

## Discussion

In this pilot study that looked at whether a video intervention in the ED would be a feasible and effective measure to prevent falls in the geriatric population, we found nearly all (92%) of participants found the interventions reasonable to implement (aim one). This suggests that most patients find certain behavioral changes, such as wearing proper footwear and hydrating more, applicable. This also follows the idea that the ED is an ideal location to approach patients and present new information, as the fall is fresh in the patients’ minds and they are more receptive to new advice [10]. It suggests that further studies aimed at ED-based education initiatives focused on behavior change and preventative interventions would be worth pursuing.

Though most patients said the interventions were reasonable, only about half of them actually implemented some of these changes at the one-month and six-month follow-ups (aim two). Some of the changes that patients undertook included focusing on balance and using walking aids. There are several possible barriers that could be affecting this lower rate of implementation: one could be memory-related. We believe that the ED is an ideal teaching environment for patients, as described above, but some patients could have the opposite response. The ED tends to be a noisy and distracting place that may affect some patients' attention while watching the video. Another barrier includes the fact that many patients underestimate the consequences of a fall. In addition, lack of reinforcement of the importance of these interventions by the patient’s doctor could be a barrier; it’s been shown that while 96% of primary care providers see the importance of fall risk assessment and interventions and think that it should be done, only 52% felt comfortable enough to do it themselves and only 14% were aware of the available CDC risk assessment tools [16]. Many patients also mentioned that they already do many of the recommendations from the video in their daily lives, and therefore said that they had not implemented anything new since the initial fall. Another barrier that is frequently cited in the literature is limited resources, for example, the inability to find transportation to get to an exercise of physical therapy class [17,18]. These barriers should be explored further to increase the implementation of fall prevention interventions.

There was not much difference between implementation at one month versus six months (57.4% to 44.7%, respectively), suggesting that once a change has been implemented, it is maintained in the long-term. This suggests that efforts should be focused on getting patients to initially change their behaviors and that those changes, once started, should be easier for patients to maintain.

Rates of recurrent falls in all patients were quite elevated at 21.1% for all participants at six months, which is expected based on previous studies stating that the one risk factor for falling is a history of prior falls [8]. Rates of return to the ED for any reason were elevated as well, at 8.5% at one month and 31.6% at six months (aim four). Reasons for ED revisits varied, from recurrent falls to very different complaints, including urinary tract infection, stroke, and a persistent cough. These rate of recurrent falls and rates of ED revisits at six months are similar to rates reported in other studies [1]. In fact, one paper studying the STEADI guidelines reported recurrent falls in 20.6% of participants and a return to the ED in 26.6% [19], while another study reported 22.6% recurrent falls and 42.6% ED revisits [20].

This study also compares rates of recurrent falls between patients who implemented some video interventions versus patients who did not. It was found that the rate of recurrent falls hovered around 20% at both one month and six months for both groups, and the difference was not statistically significant. The same can be said of perceived health status since the original fall (aim three); no significant difference was found between those who implemented the interventions and those who did not. As this is a pilot study and the sample size of patients was small, it is to be expected that no significant difference could be seen. Larger studies with more power, and with less loss to follow-up, should be conducted to see if there is any trend of significant difference in rates between the two groups.

This study has several limitations. One is the fact that this was a small sample pilot study. Another limitation is limited comprehension and retention of information and low reliability of patients in reporting changes. Several patients seemed disinterested or distracted during the video - the length of the video, the noise level in a crowded ED, and the fact that many things are happening at once in an ED (patient is awaiting imaging and results, staff members are coming in and out of rooms, patients have moved around to many different areas in the ED) could all have contributed to this distractibility, and possible low retention, despite the ED being an ideal teachable environment. Patients were given a paper copy of the specific interventions described in the video to take home, but it was noted that several patients threw the paper away or forgot it in the ED after discharge. One suggestion could be to ask the patient to repeat back three of the behavioral changes that they learned about during the intervention at the end of the session. Another potential intervention would be to have the patient view the video with a family member or caregiver for reinforcement. One last limitation is that the video may have tried to accomplish too much. Several patients noted that the video was too long or patients were noted to be distracted during the viewing. Perhaps showing patients just one intervention - wearing more secure footwear, for example - and measuring the rate of falls after implementing this one change would demonstrate a positive effect.

## Conclusions

In conclusion, geriatric falls continue to be a major problem and one that will only worsen as the general population ages. This pilot study further shows that there is a definite need for intervention and that falls continue to be a major source of morbidity and mortality for elderly patients. This study also suggests that the ED visit is an ideal teachable moment where patients are receptive to hearing about interventions, which supports previous studies. This study is also interesting in that it suggests that most patients do find behavioral interventions feasible and reasonable to implement, but only half actually make changes to their lives to reduce the risk of falling. This finding lends support to the idea that identifying and limiting barriers to implementation, including providing more easily accessible resources and reinforcing information by the patients’ primary care providers, should be a priority in future studies.

## Appendices

Appendix A - Study video

Appendix B - Patient information sheet

*Stand Up and Fight Falls *-​​​* Implementation of a Patient Education Video to Prevent Geriatric Falls*

Information for participants: *What?* We would like for you to watch a short video about the dangers of falling that will provide helpful steps to prevent you from having future falls. We would also like you to participate in a one-month and six-month follow-up phone call to determine if you have fallen again, returned to the ED, or implemented any of the changes suggested in the video. Whether or not you choose to watch this video and participate in the follow-up phone calls will not affect the care you receive in the ED.

*Why?* We hope that watching this video that provides the steps necessary to prevent falling will decrease your future fall risk. The follow-up phone calls will help us find out if the video decreased your risk of fall and if you were able to implement the fall prevention strategies suggested in the video.

*Who?* Dr. Shan Liu, MD is the local physician in charge of this research project. She can answer any questions you have. You can contact her at 617-726-4809.  You can also call Katie Davenport, MD who is a co-investigator on this study at 703-887-6444 or Blair Parry, Research coordinator, 617-724-4758 M-F 9-5 or 24/7 with questions about this research study.

*How?* We will have you view the short video on a hospital computer-on-wheels or iPad prior to your discharge from the emergency department. You will also be given a handout that will summarize the major points made in the video.

*Payment?* There is no payment for participating in this study.

*Results?* We are collecting this data for research purposes only. We will not provide you or anyone else your specific results. We will combine your results with the results of all other participants in a totally anonymous way.

*Risks?* There are no risks from participating in this research

*Benefits? *We hope participating in this study will give you the tools to prevent future falls.  Additionally, it will provide a new format for patient education in the emergency department to help prevent adverse events and recurrent falls.

*Do I have to participate?* No, your participation is completely voluntary. You have the right to decline participation.  If you agree to take part in the study but then want to drop out we will make sure that you stop the study safely.

*Problems?* If you have complaints or concerns about the research, you can call the study staff (see above).

We are required by the Health Insurance Portability and Accountability Act (HIPAA) to protect the privacy of health information obtained for research. This is an abbreviated notice, and does not describe all details of this requirement (see Partners Privacy Notice*). During this study, identifiable information about you or your health will be collected and shared with the researchers conducting the research. In general, under federal law, identifiable health information is private. However, there are exceptions to this rule. In some cases, others may see your identifiable health information for purposes of research oversight, quality control, public health and safety, or law enforcement. We share your health information only when we must, and we ask anyone who receives it from us to protect your privacy.

*Partners healthcare notice for use and sharing of protected health information

http://www.partners.org/Asets/Documents/Notices/Partners_Privacy_Policy_English.pdf

Appendix C - Questions asked after viewing video

Following the viewing of the video please ask the following questions:

1. How many times have you (the patient) fallen in the last year (including this one)? ____

2. Date of fall related to this ED presentation__________                            

3. Date of previous fall (excluding the fall related to this ED presentation) ______

4. How will you follow/implement any of the recommendations in the video? ____________

5. Are the recommendations in the video reasonable to implement? ___________________

6. How could we improve this video? ____________________________________________

7. Would you consider using a smart phone/tablet device to help you decrease your future fall risk?  ___________________________________________________________________

Appendix D - Follow-up phone calls, one month and six months

“Hello, my name is _________.  You participated in a falls study where you viewed a video on, __/__/__.  We are calling to follow up with you.   Do you remember participating in this study?   Would you be willing to participate in this follow-up call?”

Willing to participate? Yes/No

Have you had any falls since last ED visit? Yes/No

How many? ________

Have you implemented any of the interventions recommended in the video? Yes/No

If so, which one(s)? _____________

Have you returned to the ED since last visit? Yes/No

How many times? ________

Reason for visit? _________

Have you been admitted to the hospital since last visit? Yes/No

How many times? _________

Reason for visit? __________

How has your health been since your fall? Better/Same/Worse

Explain________________________-

As a reminder, the seven ways to decrease your fall risk include improving strength and balance, making home safety updates, avoiding high-risk medications, getting your vision checked, staying well hydrated, wearing appropriate footwear, and knowing what to do in case you do fall.  Do you have any questions about these recommendations?

As a reminder, we will also be conducting a brief follow up at six months.  Thank you very much for participating in this study.”

(The script for the six-month follow-up phone call is identical, except for replacing “since your last visit” with “since the one-month follow-up phone call.”)
